# Modeling colorectal cancer: A bio‐resource of 50 patient‐derived organoid lines

**DOI:** 10.1111/jgh.15818

**Published:** 2022-03-10

**Authors:** Rebekah M Engel, Thierry Jardé, Karen Oliva, Genevieve Kerr, Wing Hei Chan, Sara Hlavca, David Nickless, Stuart K Archer, Raymond Yap, Pravin Ranchod, Stephen Bell, Ann Niap, Christine Koulis, Ashley Chong, Simon Wilkins, Trevor C Dale, Andrew J Hollins, Paul J McMurrick, Helen E Abud

**Affiliations:** ^1^ Department of Anatomy and Developmental Biology Monash University Melbourne Victoria Australia; ^2^ Development and Stem Cells Program Monash Biomedicine Discovery Institute, Monash University Melbourne Victoria Australia; ^3^ Department of Surgery, Cabrini Hospital Cabrini Monash University Melbourne Victoria Australia; ^4^ Centre for Cancer Research Hudson Institute of Medical Research Melbourne Victoria Australia; ^5^ Anatomical Pathology Department Cabrini Pathology, Cabrini Hospital Melbourne Victoria Australia; ^6^ Monash Bioinformatics Platform Monash University Melbourne Victoria Australia; ^7^ Department of Epidemiology and Preventive Medicine, School of Public Health and Preventive Medicine Monash University Melbourne Victoria Australia; ^8^ European Cancer Stem Cell Research Institute (ECSCRI) Cardiff UK; ^9^ School of Biosciences Cardiff University Cardiff UK

**Keywords:** bowel cancer, colorectal, organoid, personalized medicine, tumoroid

## Abstract

**Background and Aim:**

Colorectal cancer (CRC) is the second leading cause of cancer death worldwide. To improve outcomes for these patients, we need to develop new treatment strategies. Personalized cancer medicine, where patients are treated based on the characteristics of their own tumor, has gained significant interest for its promise to improve outcomes and reduce unnecessary side effects. The purpose of this study was to examine the potential utility of patient‐derived colorectal cancer organoids (PDCOs) in a personalized cancer medicine setting.

**Methods:**

Patient‐derived colorectal cancer organoids were derived from tissue obtained from treatment‐naïve patients undergoing surgical resection for the treatment of CRC. We examined the recapitulation of key histopathological, molecular, and phenotypic characteristics of the primary tumor.

**Results:**

We created a bio‐resource of PDCOs from primary and metastatic CRCs. Key histopathological features were retained in PDCOs when compared with the primary tumor. Additionally, a cohort of 12 PDCOs, and their corresponding primary tumors and normal sample, were characterized through whole exome sequencing and somatic variant calling. These PDCOs exhibited a high level of concordance in key driver mutations when compared with the primary tumor.

**Conclusions:**

Patient‐derived colorectal cancer organoids recapitulate characteristics of the tissue from which they are derived and are a powerful tool for cancer research. Further research will determine their utility for predicting patient outcomes in a personalized cancer medicine setting.

## Introduction

With an estimated 1.8 million new cases and 881 000 deaths in 2018, colorectal cancer (CRC) is the third most commonly diagnosed and the second leading cause of death worldwide.^1^ To improve outcomes and reduce CRC‐related deaths, we must develop new strategies for the detection and treatment of disease. Treatment for patients with advanced stage cancer have traditionally relied on the prescription of chemotherapeutic drugs, selected based on the organ of origin, histological features of the tumor, and the tumor‐node‐metastasis staging.^2^ However, we know that even when controlled for similar preoperative clinical features, patients respond differently to the same treatment and this presents a key challenge in effective administration of current and new therapeutic strategies.

With the advent of personalized cancer medicine, focus has now shifted to the development of treatment strategies based on the individual characteristics of the patients' tumor to provide the best possible outcome with the fewest side effects. While molecular profiling has been used to identify some therapeutic options, it fails to provide a functional assessment of tumor response. Innovative model systems such as organoid culture promise to improve the translation of preclinical studies by demonstrating patient‐specific drug sensitivity, even in the absence of targetable biomarkers.

Organoids are complex, three‐dimensional cultures derived from stem cells that contain organ‐specific cell types and have the ability to self‐organize.^3^, ^4^ Long‐term culture of epithelial organoids established from human colonic and neoplastic tissue was first described by Sato *et al*. in 2011, providing a robust, near‐physiological model that retains key genetic and phenotypic features of the tissue from which it is derived.^4^, ^5^ In contrast to patient‐derived xenograft mouse models, organoids can be cultured with high efficiency from small amounts of tissue in a short period of time (< 4 weeks). There is also increasing evidence that patient‐derived tumor organoids can mimic the treatment responses of patients in the clinic, albeit on relatively small patient cohorts.^6^, ^7^, ^8^, ^9^ This technology creates a platform that has the potential to not only guide treatment choice based on standard‐of‐care therapies but also identify novel treatment strategies for chemorefractory patients who fail standard therapies and have no further treatment options available, as demonstrated in a recent study by Narasimhan *et al*.^7^

For organoid‐based technology to be an effective screening strategy to determine patient response, organoids need to be efficiently established from different sites, recapitulate the features of the tumors from which they are derived, and be amenable to freeze/thaw procedures to enable drug testing in different time frames. In this report, we examine the derivation of 50 patient‐derived colorectal cancer organoids (PDCOs) established in our study and review the potential utility of this model system for predicting patient response to various therapeutic agents.

## Methods

### Ethics and consent

This study was conducted in accordance with the Declaration of Helsinki, and the protocol was approved by the Cabrini Research Governance Office (CRGO 04‐19‐01‐15) and the Monash Human Research Ethics Committee (MHREC ID 2518). Patient recruitment was led by the colorectal surgeons in the Cabrini Monash University Department of Surgery. Tissue was obtained from treatment naïve patients diagnosed with CRC undergoing surgical resection at the Cabrini Hospital, Malvern, Australia. All patients provided written informed consent.

### Patient database

Patients undergoing abdominal surgery for colorectal neoplasia at Cabrini Hospital are included in the prospectively maintained, clinician‐led Cabrini Monash Colorectal Neoplasia Database.^10^ This dataset has been adopted in a minimum dataset format as the Binational Colorectal Cancer Audit of the Colorectal Surgical Society of Australia and New Zealand (https://cssanz.org/bcca‐database/). Up to 350 parameters are recorded throughout the patient management process from time of presentation, including details of diagnosis, surgery, pathology, treatment, and follow up (up to 5 years). Each of the patients who have donated tissue for the colorectal organoid study are included on the database, and this provides comprehensive clinical records and treatment outcomes that can be correlated to the characteristics of their own tissue‐derived organoid lines.

### Establishing organoids

Colorectal cancer tissue specimens were cut into 5 mm pieces and washed eight times with 1X phosphate‐buffered saline (PBS) supplemented with antibiotics. Tissue fragments were digested with 0.125 mg/mL dispase type II (Sigma, St Louis, MO, USA) and 1mg/mL collagenase A (Roche Diagnostics, Mannheim, Germany) at 37°C for 30 min and then mechanically dissociated by repetitive pipetting in cold PBS. Cancer tissue fragments were allowed to settle by gravity before supernatant was collected and pelleted by centrifugation at 240*g* for 5 min at 4°C. The isolated cells/fragments were passed through a 70 μm cell strainer (Corning, NY, USA), centrifuged, and resuspended in Matrigel (Corning).

Patient‐matched normal colonic tissue was collected from the margins of the resected specimen. After removing underlying muscle layers and adipose tissue with surgical scissors, normal colonic tissue was cut into 5 mm pieces and washed eight times in cold chelation buffer (distilled water with 5.6 mmol/L Na_2_HPO_4_, 8.0 mmol/L KH_2_PO_4_, 96.2 mmol/L NaCl, 1.6 mmol/L KCl, 43.4 mmol/L sucrose, and 54.9 mmol/L d‐sorbitol). Tissue fragments were then incubated for 45 min at 4°C in 4 mM EDTA chelation buffer. Intestinal crypts were released from colonic tissues by mechanical dissociation, pipetting tissue fragments in cold chelation buffer. Tissue fragments were allowed to settle by gravity before supernatant was collected and pelleted by centrifugation at 240*g* for 5 min at 4°C. The crypt isolation was passed through a 100 μm cell strainer (Corning), centrifuged, and resuspended in Matrigel.

Matrigel containing normal colonic crypts and cancer cell clusters were seeded into 24‐well tissue culture plates (Thermo Scientific Nunc, Foster City, CA, USA) and allowed to polymerize for 10 min at 37°C. The normal colonic crypts were overlaid with 500 μL of culture medium composed of advanced Dulbecco's Modified Eagle Medium/F12 supplemented with 1X B27, Glutamax, 10 mM HEPES (all from Gibco, Waltham, MA, USA), 100 μg/mL Primocin (InvivoGen, San Diego, CA, USA), 50 ng/mL recombinant human EGF (Peprotech, Rochy Hill, NJ, USA), 10 nM Gastrin (Sigma), 500 nM A83‐01 (Tocris Bioscience, Bristol, UK), 10 μM SB202190 (Sigma), 1.25 mM *N*‐acetylcysteine (Sigma), 10 mM nicotinamide (Sigma), 100 ng/mL recombinant human Noggin (Peprotech) or 10% Noggin conditioned media, 20% R‐spondin 1 conditioned media, and 50% WNT3A conditioned media. Colon cancer cells were cultured with the same media minus WNT3A conditioned media. Following initial seeding of the cultures, 10 μM Y‐27632 dihydrochloride kinase inhibitor (Tocris Bioscience) and 2.5 μM CHIR99021 (normal tissue only; Stemgent, Cambridge, MA, USA) were also added to the media.

### Histological sections

Primary tissue samples were fixed in 4% paraformaldehyde and embedded in paraffin blocks. Mature organoids were fixed in 4% paraformaldehyde before being dissociated from the Matrigel. Organoids were collected into a tube and gently centrifuged before being embedded into low melting agarose (2% in PBS) or HistoGel (Thermo Scientific, Kalamazoo, MI, USA). The blocks were processed before being embedded into paraffin. Sections (4 μm thick) of both primary tissue and patient‐matched organoids were subjected to routine hematoxylin and eosin staining.

### Immunohistochemistry

Immunohistochemical staining was performed as previously described.^11^, ^12^ Briefly, 4 μm paraffin sections were deparaffinized in histosol and rehydrated in graded alcohols. Antigen retrieval was performed by incubating the slides for 30 min at 98°C in citrate buffer (Target Retrieval Solution S1699, Dako, Burlingame, CA, USA). Slides were incubated with Peroxidase Blocking Solution (Dako, S2023) for 10 min, followed by protein block (Dako, X0909) for 10 min. Sections were incubated for 1 hour at room temperature with the primary antibody diluted in antibody diluent (Dako, S0809). The following antibodies were used: anti‐Cytokeratin 20 (Abcam, ab64090, 1:200), anti‐Cytokeratin 7 (Abcam, ab183344, 1:200), and anti‐caudal type homeobox 2 (CDX2) (Abcam, ab76541, 1:1000). For the detection of primary antibodies, sections were exposed to anti‐rabbit horseradish peroxidase coupled antibody (Dako, K4003) for 1 hour at room temperature. Peroxidase activity was detected with the 3,3′‐diaminobenzidine liquid substrate (Dako, K3468). Sections were counterstained with hematoxylin (Dako, S3301), dehydrated and mounted. Slides were scanned on the Aperio Scanscope AT Turbo (Leica Biosystems, Wetzlar, Germany). Positive staining was assessed by expected location (nuclear/cytoplasmic/membrane) and abundance for each marker, that is, strong and diffuse nuclear staining of CDX2+, cytoplasmic, and/or cell membrane staining of cytokeratin 20 (CK20) and cytokeratin 7 (CK7). No primary controls were included for each marker. CRC tissue sections were included as positive controls for CDX2 and CK20, with breast cancer tissue sections included as a positive control for CK7.

### Whole exome sequencing

DNA from primary tumor tissue, PDCOs, and normal reference tissue/organoids was extracted using the QIAamp® Fast DNA Tissue Kit (Qiagen). Whole exome sequencing (WES) was conducted using the Agilent SureSelect Exome V6 library kit to prepare the libraries and sequenced on the Illumina HiSeq platform and sequenced to 50X (normal tissue and blood reference) and 100X (tumor) average coverage, with 2× 150 bp paired‐end reads (GENEWIZ from Azenta Life Sciences, NJ, USA).

### Bioinformatics analysis

Reads were initially aligned to the Hg19 human genome reference using the Dragen v2.4.5 Illumina pipeline by the sequencing facility (GENEWIZ). Reads around variants were realigned more accurately with GATK^13^ tools RealignerTargetCreator and IndelRealigner, followed by initial variant calling with GATK HaplotypeCaller and filtering out very unlikely variants using GATK VariantFiltration (filter expression was “QD < 2.0||FS > 60.0||MQ < 40.0||MQRankSum < −12.5||ReadPosRankSum < −8.0||SOR > 4.0” for SNPs and “QD < 2.0||FS > 200.0||ReadPosRankSum < −20.0||SOR > 10.0” for indels). Read quality values were then recalibrated for the sequencing run, taking these variants into account, using the GATK BaseRecalibrator tool before finally calling confident somatic variants, copy number variations, and clonal compositions using SuperFreq,^14^ subtracting out germline variants using each tumor line's paired normal sample. PDCOs, primary tumor, and normal tissue samples were verified as matching by collecting all SNPs called as homozygous (genotype call quality > 98) by GATK HaplotypeCaller, then filtering out variants with a study‐wide allele count of < 3 or sequencing depth < 21. The Jaccard (binary) distance between each pair of samples was then calculated to verify that samples from the same patient had significantly more variants in common between them than in unrelated samples.

## Results

### Establishment of a living colorectal cancer organoid bio‐resource

Tissue samples were obtained from consenting patients undergoing surgical resection for the treatment of colorectal cancer at Cabrini Hospital Malvern, between August 2015 and February 2019. In this study, inclusion criteria specified that tissue specimens were collected from patients who were treatment‐naïve at the time of surgery, with subsequent pathological confirmation of primary colorectal adenocarcinoma. Patients with benign neoplasia were not included in this report.

A summary of patient and tumor characteristics for this cohort are presented in Table 1 (described in full, Table S1). The median patient age was 72 years with a range from 27 to 92 years, in equal proportions of male to female. The pathological tumor type was mostly adenocarcinoma (86%), with mucinous adenocarcinoma making up the remainder of the cohort (14%). No patients with signet ring cell carcinomas were recruited into this study. Tumor‐node‐metastasis staging confirmed the inclusion of tumors from each of the four stages of CRC. The majority of the PDCOs were established from tumors defined as moderately differentiated (64%); however, PDCOs were also been established from both well (8%) and poorly (28%) differentiated tumors. Similarly, both mismatch repair (MMR) proficient (82%) and MMR deficient (dMMR; 18%) tumors were successfully established from this cohort.

**Table 1:** Patient and tumor characteristics

Variable		
	Colon	Rectal^†^
Number of patients	40 (80%)	10 (20%)
Gender, *n* (% of total)		
Female	22 (44%)	3 (6%)
Male	18 (36%)	7 (14%)
Age, *n* (% of total)		
< 50 years	2 (4%)	2 (4%)
50–59 years	4 (8%)	2 (4%)
60–69 years	8 (16%)	1 (2%)
70–79 years	12 (24%)	4 (8%)
80–89 years	12 (24%)	1 (2%)
≥ 90 years	2 (4%)	0 (0%)
Tumor type, *n* (% of total)		
Adenocarcinoma	33 (66%)	10 (20%)
Mucinous adenocarcinoma	7 (14%)	0 (0%)
Overall stage, *n* (% of total)		
I	6 (12%)	3 (6%)
II	12 (24%)	2 (4%)
III	16 (32%)	3 (6%)
IV	6 (12%)	2 (4%)
Differentiation, *n* (% of total)		
Well differentiated	2 (4%)	2 (4%)
Moderately differentiated	25 (50%)	7 (14%)
Poorly differentiated	13 (26%)	1 (2%)
MMR status, *n* (% of total)		
MMR proficient	31 (62%)	10 (20%)
MMR deficient	9 (18%)	0 (0%)

^†^ Rectal denotes any tumor located at ≤ 15 cm from anal verge.

**Figure 1:**
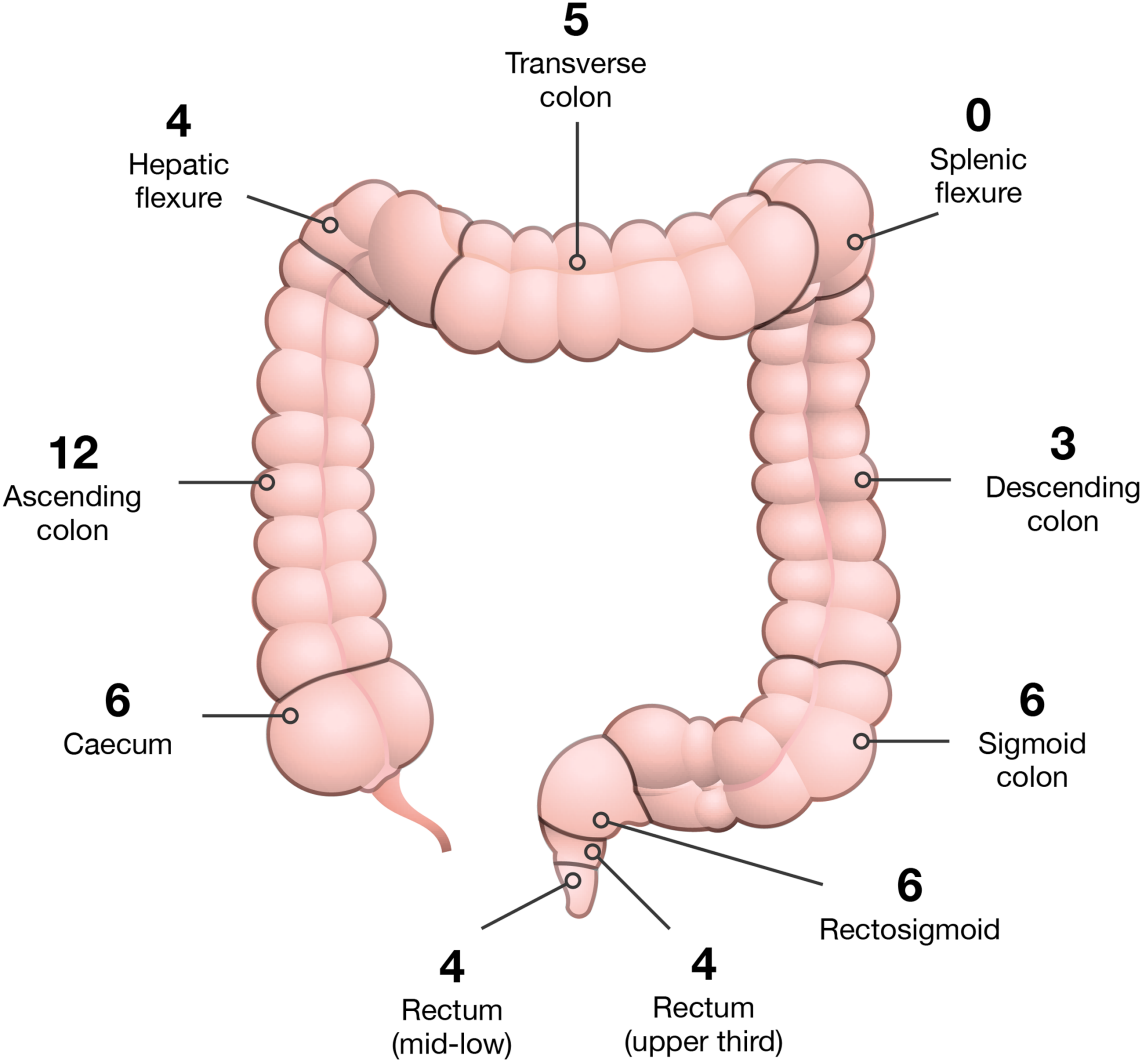
Anatomical location of primary colorectal tumors from which patient‐derived colorectal cancer organoids were generated for the bio‐resource. Patient‐derived colorectal cancer organoids have been successfully established from all 10 regions, excluding the splenic flexure. [Color figure can be viewed at wileyonlinelibrary.com]

Right‐sided or proximal CRCs arise from the caecum to transverse colon, whereas left‐sided or distal CRCs arise from splenic flexure to rectum. The ratio of established PDCOs in relation to sidedness is 27 RHS: 23 LHS. Figure 1 illustrates the primary tumor location throughout the colon and rectum for the first 50 patient lines generated for our bio‐resource, with successful establishment of PDCOs from all 10 regions, excluding the splenic flexure.

Adjacent normal tissue was also collected from each patient at the time of resection with a normal organoid line successfully established from isolated intestinal crypts for 50% of the cohort (*n* = 25). Additionally, three stage IV patients who underwent synchronous resections have PDCOs established from both their primary and metastatic tumors. These include liver (*n* = 4; three lines from one patient) and peritoneal (*n* = 1) metastatic tumor organoid lines. Over time, we have optimized our reagents and culture techniques, with our success rate for establishing tumoroids now ~80% and for patient‐matched normal organoids it is ~100%.

### Histopathological characteristics are maintained in patient‐derived colorectal cancer organoids

**Figure 2:**
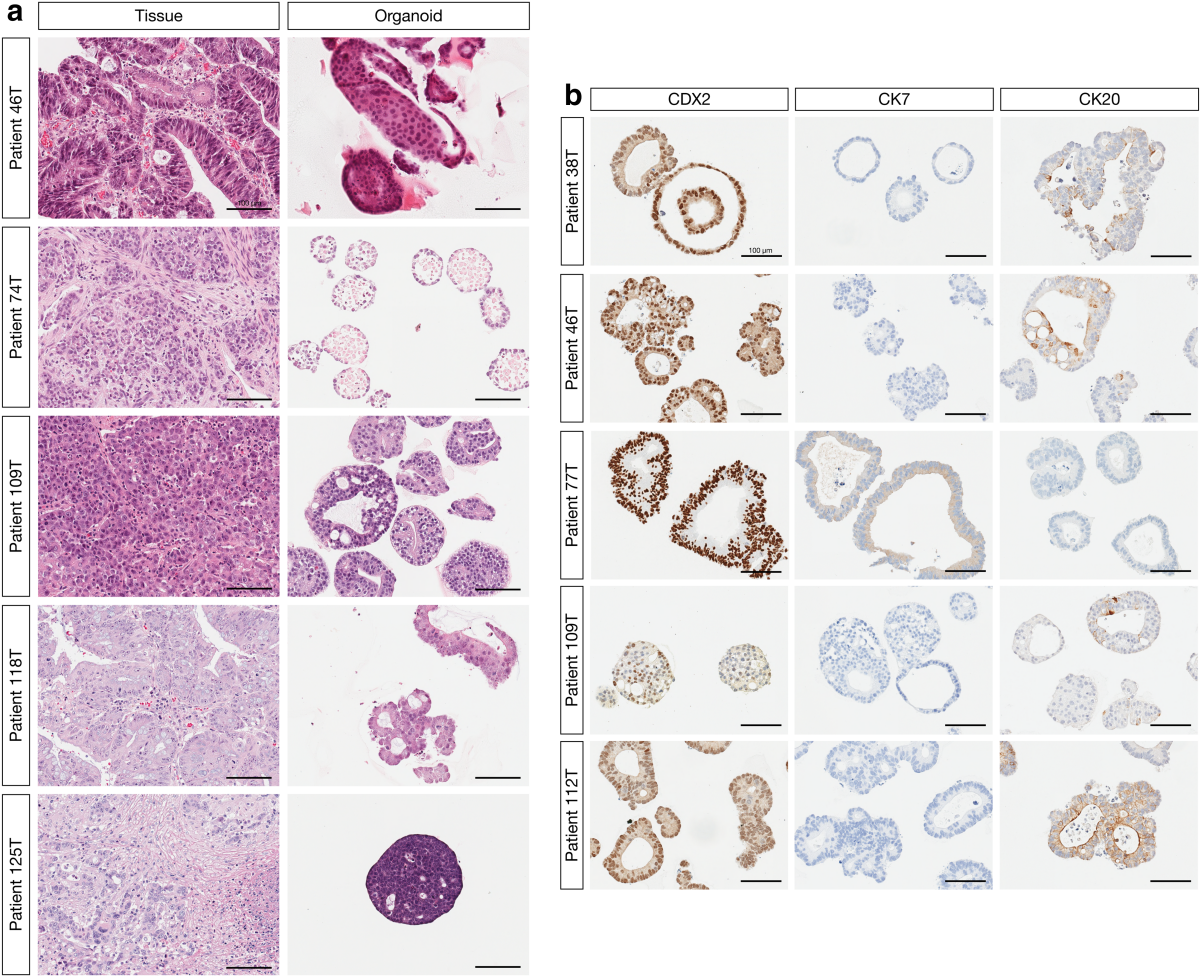
(a) PDCOs recapitulate the histopathological characteristics of the primary tumors. Hematoxylin and eosin (H&E) staining of sectioned tissue from primary colorectal adenocarcinoma (left panel) and patient‐derived colorectal cancer organoids derived from the same tissue (right panel). Scale bar, 100 μm. (b) Immunohistochemical detection of markers used in the differential diagnosis of colorectal cancer, CDX2, CK7, and CK20 in patient‐derived colorectal cancer organoids. Scale bar, 100 μm. [Color figure can be viewed at wileyonlinelibrary.com]

Hematoxylin and eosin staining was performed on paraformaldehyde‐fixed and paraffin‐embedded primary tumor tissue and patient‐matched PDCOs. Histological analysis was conducted independently by two trained pathologists to assess cellular architecture and cytological features including nuclear shape, degree of nuclear atypia and pleomorphism, amount of cytoplasm, presence or absence of intracytoplasmic mucin, with the profile of the primary tumor generally well maintained in PDCOs (representative images Fig. 2a). PDCO cells are generally smaller and more cuboidal, but otherwise show broad conservation of tumor cell morphology. PDCOs were further validated through the immunohistochemical analysis of markers that are commonly used in the differential diagnosis of CRC; CDX2, a transcription factor critical for intestinal development that is highly expressed in normal and neoplastic intestinal epithelium,^15^, ^16^ CK20, normally expressed in gastrointestinal epithelium, Merkel cells and the urothelium,^17^, ^18^ and CK7, detected in normal tissue and tumors of the lung, breast, ovary, biliary tract, and endometrium^19^, ^20^ (representative images Fig. 2b; refer also to Figure S1). The typical CK20+/CK7− immunophenotype that is highly characteristic of colorectal carcinomas was observed in the majority of PDCOs.

### Key molecular characteristics are retained by patient‐derived colorectal cancer organoids

**Figure 3:**
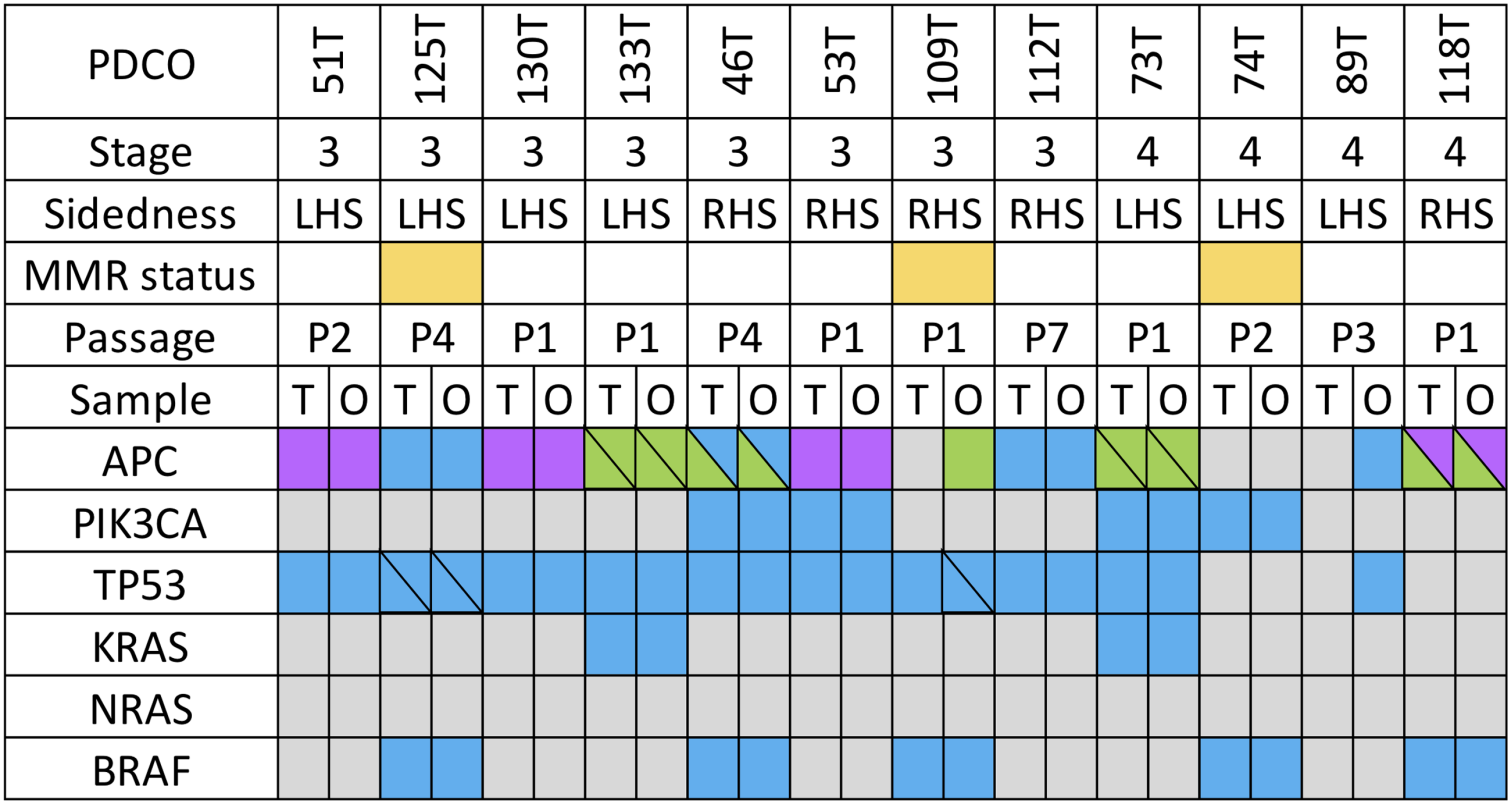
Patient clinical information for the corresponding PDCO on overall stage, sidedness of primary tumor and MMR status. Genomic characterization was performed using WES with alterations in key driver mutations compared in the patient's bulk tumor sample and the associated PDCOs. The majority of alterations were identical with the exception of a PIK3CA mutation in 53 T, and an APC and TP53 mutation in PDCOs 109 and 89 T that were identified in the PDCOs, but not in the bulk tumor specimen. Further details provided in Table S2. PDCO, patient‐derived colorectal cancer organoid. T = Primary tumor specimen; O = patient‐derived cancer organoid; yellow = mismatch repair deficient; grey = no alteration; purple = nonsense; blue = nonsynonymous; green = frameshift. [Color figure can be viewed at wileyonlinelibrary.com]

**Figure 4:**
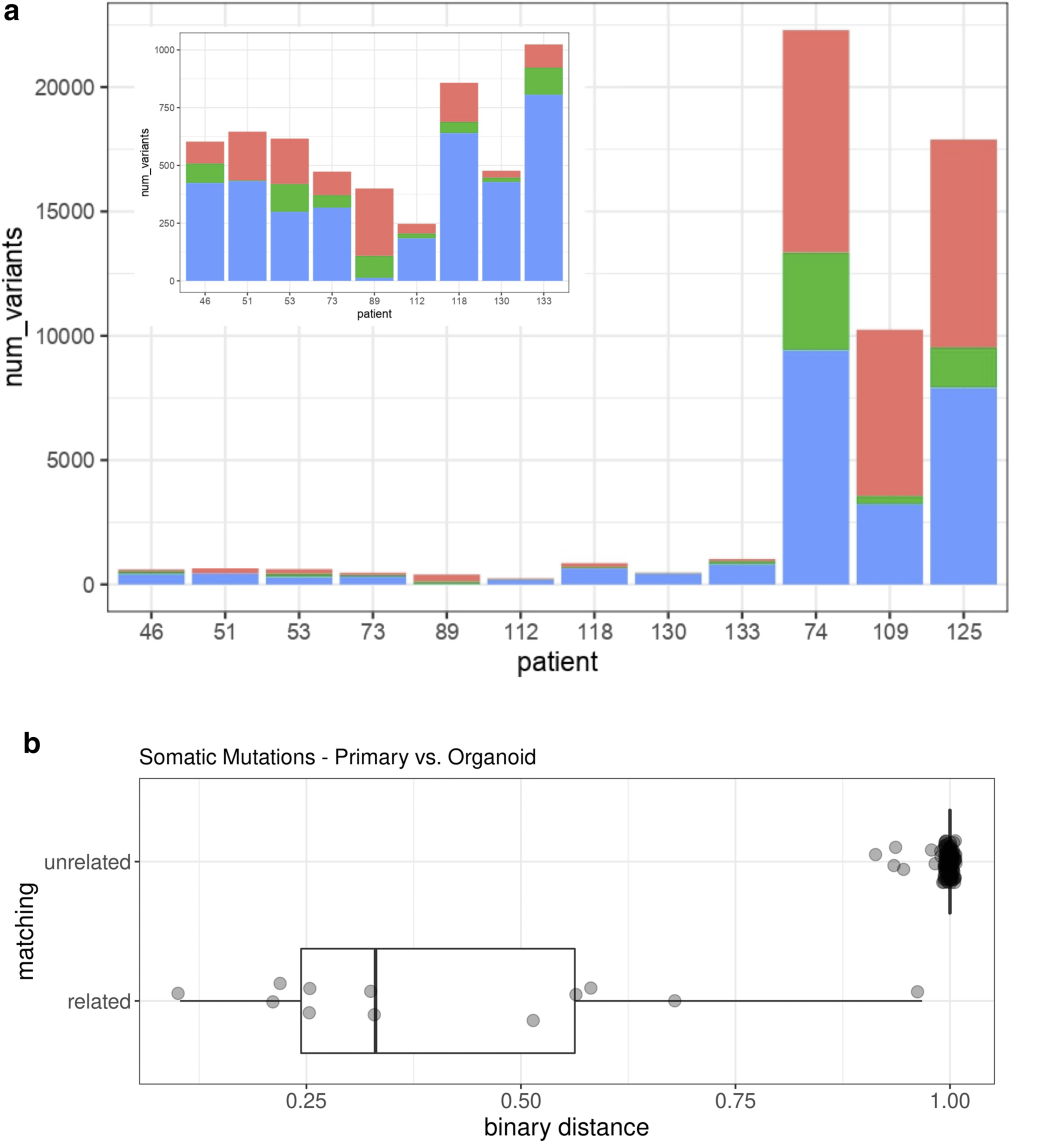
(a) Total number of somatic variants present in PDCOs and matched primary tumor, aligned to the Hg19 human genome reference. Normal reference was provided by either patient‐matched adjacent normal tissue or organoids generated from patient‐matched adjacent normal tissue, with the exception of 133 T, for which likely germline mutations were filtered out using SuperFreq's germlineLike flag. Somatic variants specific to the PDCO (coral), primary tumor (green), or shared (blue) are shown. PDCOs derived from dMMR patients (74, 109, and 125 T) have significantly higher numbers of variants (inset provided for scale). Group: coral = organoid; green = primary; blue = shared. (b) Box‐plot of Jaccard distances of high‐confidence somatic variant sets in all primary tumor/PDCO pairwise combinations sequenced in this study. Unrelated PDCO/tumor pairs (top) had a very small proportion of somatic variants in common unlike parental‐tumor/derived organoid pairs (bottom). [Color figure can be viewed at wileyonlinelibrary.com]

A cohort of our PDCOs (*n* = 12) were characterized by WES. DNA from PDCOs with passage numbers ranging from P1 to P7 and patient‐matched primary tumor tissue were sequenced. A normal reference was provided by either patient‐matched adjacent normal tissue or organoids generated from patient‐matched adjacent normal tissue. WES was conducted using the Illumina PE150 platform and sequenced to 50X (normal tissue/organoid reference) and 100X (tumor/tumoroid) average coverage. Key driver mutations that confer survival advantage and drive cancer progression were examined (Fig. 3). Additionally, somatic variants in the PDCOs and the matched primary tumor were identified as either shared by both the PDCO and primary tumor from which it was derived, or specific to each sample (Fig. 4a). The patient‐matched PDCO and primary tumor samples were also verified to have significantly more variants in common between them than in unrelated samples (Figs 4b and S2). The total number of somatic variants varied widely between each PDCO, with those derived from patients with deficient mismatch repair (dMMR) having much higher overall numbers of variants (74 T, 109 T and 125 T). PDCOs, primary tumor, and normal tissue samples were verified to have originated from the same patient, as described (Fig. S3). Overall, the PDCOs remain highly representative of the primary tumor tissue with high level of concordance in the driver mutations observed. These results were also highly concordant with colorectal gene panel testing (*BRAF*, *KRAS*, *NRAS*, and *PIK3CA*) undertaken by the hospital pathology laboratory using formalin‐fixed paraffin‐embedded tissue (*n* = 4). Interestingly, we did identify somatic variants in key driver genes for PDCOs 53, 109, and 89 T that were not detected in the primary tumor specimen. We observe maintenance of intratumoral heterogeneity of genetic mutations in the PDCOs, with multiple clones present following short‐term culture (data not shown).

### Phenotypic characteristics of the tumor are maintained across passages and freeze–thaw cycles

**Figure 5:**
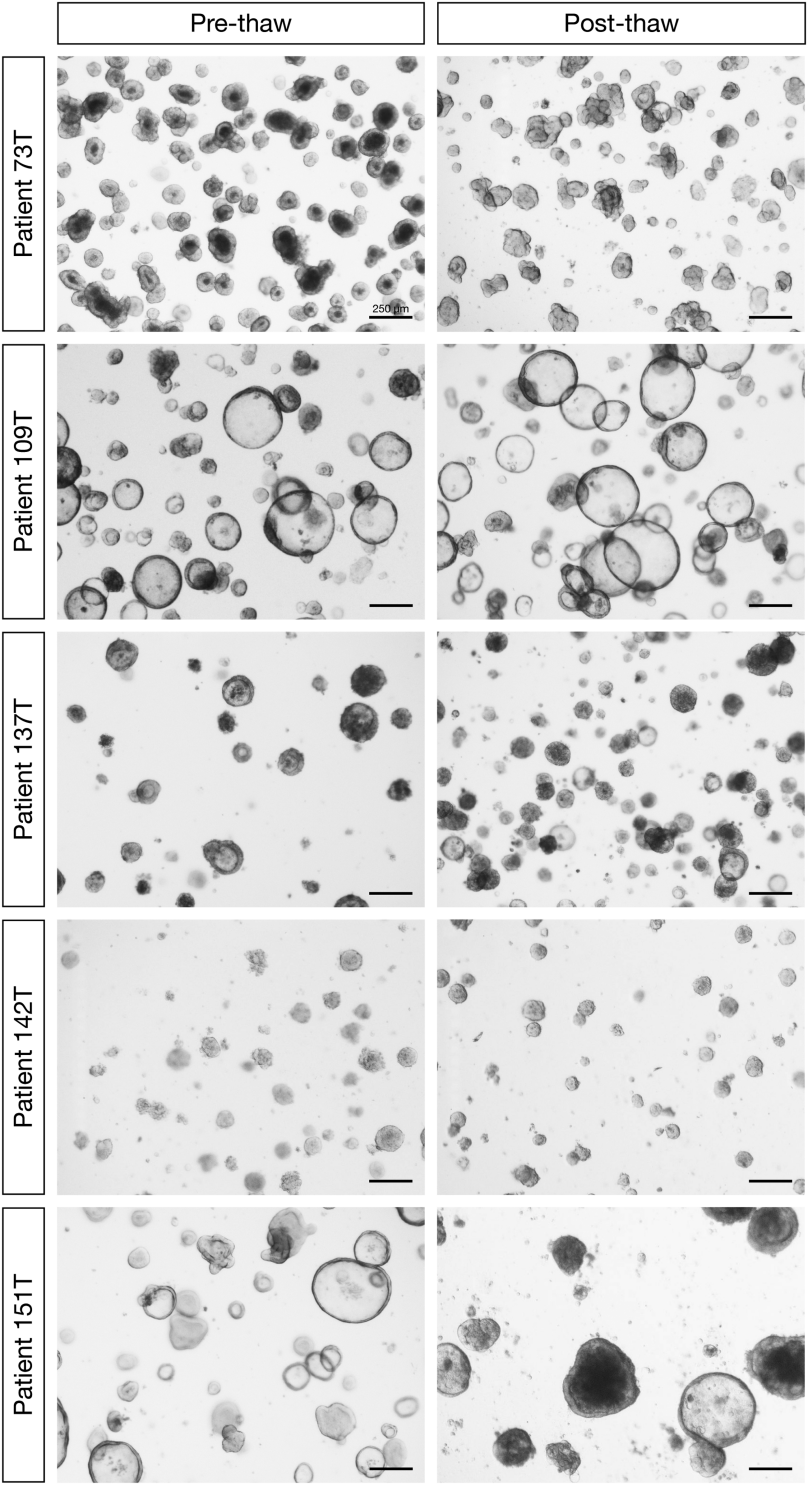
Conservation of phenotypic features following a freeze/thaw process in PDCOs, with bright‐field images of established PDCOs prior to cryopreservation (left panel) and after re‐establishment and passaging post‐thawing (right panel). Scale bar, 250 μm.

Organoid lines have great utility as a model system of disease as they can be expanded indefinitely as well as frozen and thawed in a similar way to conventional two‐dimensional cell lines. Importantly, fundamental characteristics are maintained over passages and following freeze–thaw cycles. Representative bright‐field images of PDCOs taken prior to cryopreservation and following thawing are shown in Figure 5. PDCOs maintain key phenotypic characteristics observed upon initial establishment of these lines (Fig. 5, left panel) with subsequent images captured following as many as five passages and at least one freeze–thaw cycle (Fig. 5, right panel).

## Discussion

Despite significant progress in prevention, diagnosis and treatment strategies, CRC remains a leading cause of death worldwide.^1^ Over many decades, the use of model systems of disease including classical cell lines and animal models has advanced our understanding of the complexities of CRC elucidating key cell signaling pathways, genetics and epigenetics, and the identification of potential drug targets. However, there are numerous limitations within these systems including expense, lack of cellular heterogeneity, ethical issues and failure to recapitulate human pathophysiology. With nine out of ten attempts to bring a product to market failing during the clinical trial phase, the cancer models on which these studies are predicated must poorly recapitulate the human condition.^21^ The development of 3D organoid technology has created a more functionally relevant model that retains much of the complexity and heterogeneity of the primary tissue. Importantly, as demonstrated in this and other studies, organoids remain both phenotypically and genetically representative of the tissue from which they are derived.^5^, ^22^, ^23^ These self‐organizing organotypic cultures can be established with high efficiency, expanded and cryopreserved, thus allowing the creation of a living biobank.^22^

To date, there have been a number of organoid biobanks established from patients with CRC, including organoids from both primary and metastatic lesions.^5^, ^24^, ^25^, ^26^ As in our own study, these biobanks include PDCOs derived from a range of primary tumor sites, histological subtypes, clinical stages, and genetic profiles. This creates a novel platform for both discovery and translational research. Due to the intratumoral and intertumoral heterogeneity observed in CRC, it is difficult to assess the extent to which these biobanks represent the broader patient population. However, given the intrinsic heterogeneity of PDCOs relative to the native tumor, and the significant impact this has on a patient's ability to respond to therapy, their use in predicting efficacy of standard‐of‐care and novel drugs in a personalized cancer care setting has gained significant interest.

In a proof of concept study, Vlachogiannis *et al*. were the first to demonstrate that PDCOs derived from treatment‐naïve metastatic colorectal and gastroesophageal tumors were able to recapitulate patient response, reporting a positive predictive value of 88% and a negative predictive value of 100%.^27^ Following on from this, several other small‐scale studies have demonstrated the potential of organoid technology to translate from bench to bedside, with PDCOs effectively mimicking the treatment response observed in the tumors of patients.^6^, ^7^, ^8^, ^9^ Organoids also hold much promise for novel drug discovery, enabling rapid assessment of efficacy and toxicity. A study by Fiore *et al*. demonstrated that Rimonabant, a compound capable of inactivating Wnt signaling, was effective in reducing proliferation in CRC organoids with no evidence of toxicity when treating organoids derived from healthy colon epithelium.^28^ Another study reported a strong cytotoxic effect on CRC organoids from a compound originally derived from a marine sponge.^29^ Further studies are required to determine the effective translation of these findings into the clinic.

Molecular profiling of PDCOs may be useful in identifying mechanisms of treatment resistance or indeed for the identification of targetable mutations. In this study, we have shown that key molecular alterations are maintained in PDCOs and are representative of the primary specimen, as has been previously reported.^7^, ^27^, ^30^, ^31^ Interestingly, PDCOs derived from dMMR patients continue to acquire variants at a high rate in culture; therefore, it remains to be determined how long these PDCOs continue to be representative of the primary tumor. Outside of this, minor differences should be expected due to sampling error or, as was the case for some of our samples, mutations were detectable but were below the confidence filter thresholds set by SuperFreq. This is consistent with previous findings examining genomic intratumor heterogeneity in CRC.^32^ Combining genomics and functional screening of PDCOs could be used to prospectively guide prescription of off‐label therapies. This was demonstrated in a study screening PDCOs against a panel of 25 drugs, with responses to therapy associated with the genetic alterations present in the primary tumor and recapitulated in the organoid.^22^ A more recent study by Narasimhan and colleagues resulted in treatment change for two patients following genomic and drug profiling of organoids derived from metastatic peritoneal tumors.^7^ This study demonstrated the clinical utility of organoids for prospective drug sensitivity testing, with parallel next‐generation sequencing and *ex vivo* medium‐throughput drug panel testing being completed in an 8‐week timeframe. Creating a platform for drug repurposing and de novo drug discovery such as this is particularly pertinent for patients who have failed standard therapies and exhausted routine treatment options.

We have demonstrated the conservation of phenotypic features following a freeze/thaw process in PDCOs. With the ability to preserve a living replicate of a patients' own tumor that can be revitalized at any time, PDCOs have the utility to enable testing of new and emerging treatments as they are developed. This will be a particularly useful tool not only for drug discovery but also to perform *in vitro* testing for the efficacy of cutting edge treatments for patients who develop local or distant recurrence after a period of time.

As with all models, organoids are not without limitation. Organoid culture lacks important elements of the tumor microenvironment including stroma, blood vessels, and immune cells. Some studies have investigated the role of coculture in order to incorporate these components.^33^, ^34^, ^35^, ^36^ Despite this, organoid technology has created a physiologically relevant *in vitro* model for CRC and is a promising platform for the development of personalized cancer medicine, with high establishment rates from patient tissue and the ability to expand and screen PDCOs in a clinically relevant timeframe. Given that no *one* model can accurately recapitulate the human condition, a combination of approaches that are complementary will likely yield the most insight into this complex condition. In terms of limitations within our own study, while our bio‐resource is largely representative of the broader patient cohort, we cannot determine if all subtypes are represented. It is likely that rare subtypes are not represented in this collection of organoids.

In summary, this study demonstrates the feasibility of developing organoid models in CRC. There is clear demonstration that key histopathological, molecular, and phenotypic characteristics are retained from the original tumor specimens in the PDCOs. Further avenues of research including subjecting PDCOs to standard and novel treatments is currently being explored and hold the promise of both truly personalized therapy as well as a pathway to new treatment paradigms.

## Supporting information




**Figure S1.** Patient‐derived colorectal cancer organoids stained with markers used in the differential diagnosis of colorectal cancer, CDX2 (left), CK7 (second from left) and CK20 (second from right) as well as H&E staining (right). Scale bar, 100 μm.

**Figure S2.** Heat map of sample‐to‐sample distances comparing high‐confidence somatic variant calls for all PDCOs and primary tumor tissues sequenced in this study. The Jaccard distance between each pair of samples was calculated to verify that organoids had more somatic variants in common with their parental tumors than other organoid or tumor samples.

**Figure S3.** Heat map of sample‐to‐sample distances comparing high‐confidence homozygous variant calls for all PDCOs, primary tumor tissue and normal tissues/organoids sequenced in this study. The Jaccard distance between each pair of samples was calculated to verify that samples from the same patient had more variants in common between them than in unrelated pairs of samples.

**Table S1.** Patient clinical characteristics for the patient‐derived cancer organoids.

**Table S2.** Somatic variants identified in patient‐derived cancer organoids.
