# Supplementary figures and images for: Modeling colorectal cancer: A bio‐resource of 50 patient‐derived organoid lines
Source: J Gastroenterol Hepatol. 2022 Mar 10;37(5):898–907. doi: 10.1111/jgh.15818 (PMC10138743; doi:10.1111/jgh.15818)

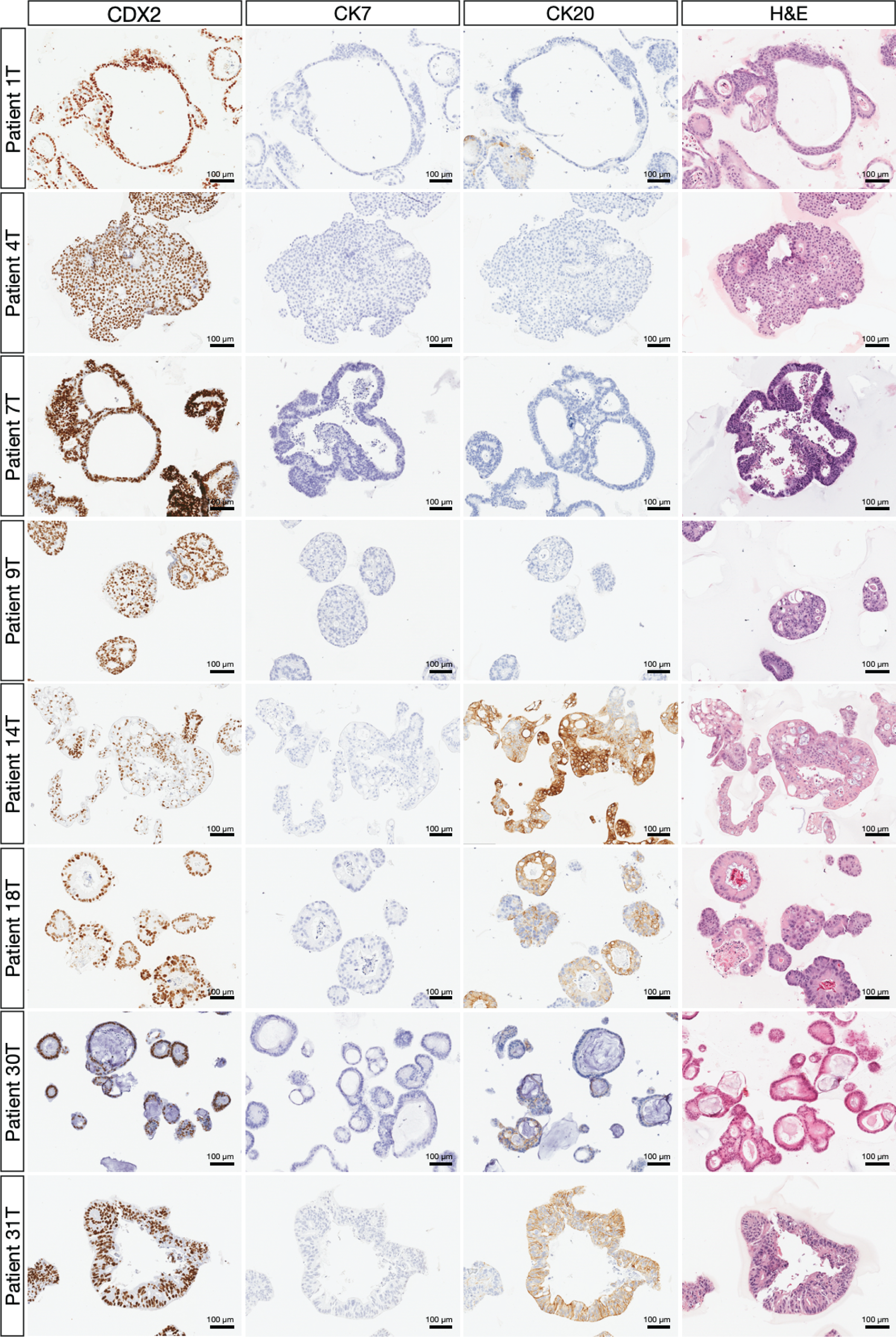

Supplement: Supplementary file 1 — Figure S1. Patient‐derived colorectal cancer organoids stained with markers used in the differential diagnosis of colorectal cancer, CDX2 (left), CK7 (second from left) and CK20 (second from right) as well as H&E staining (right). Scale bar, 100 μm. [file JGH-37-898-s002.zip › JGH_15818_Supplementary_Figure_1_Page1.png]

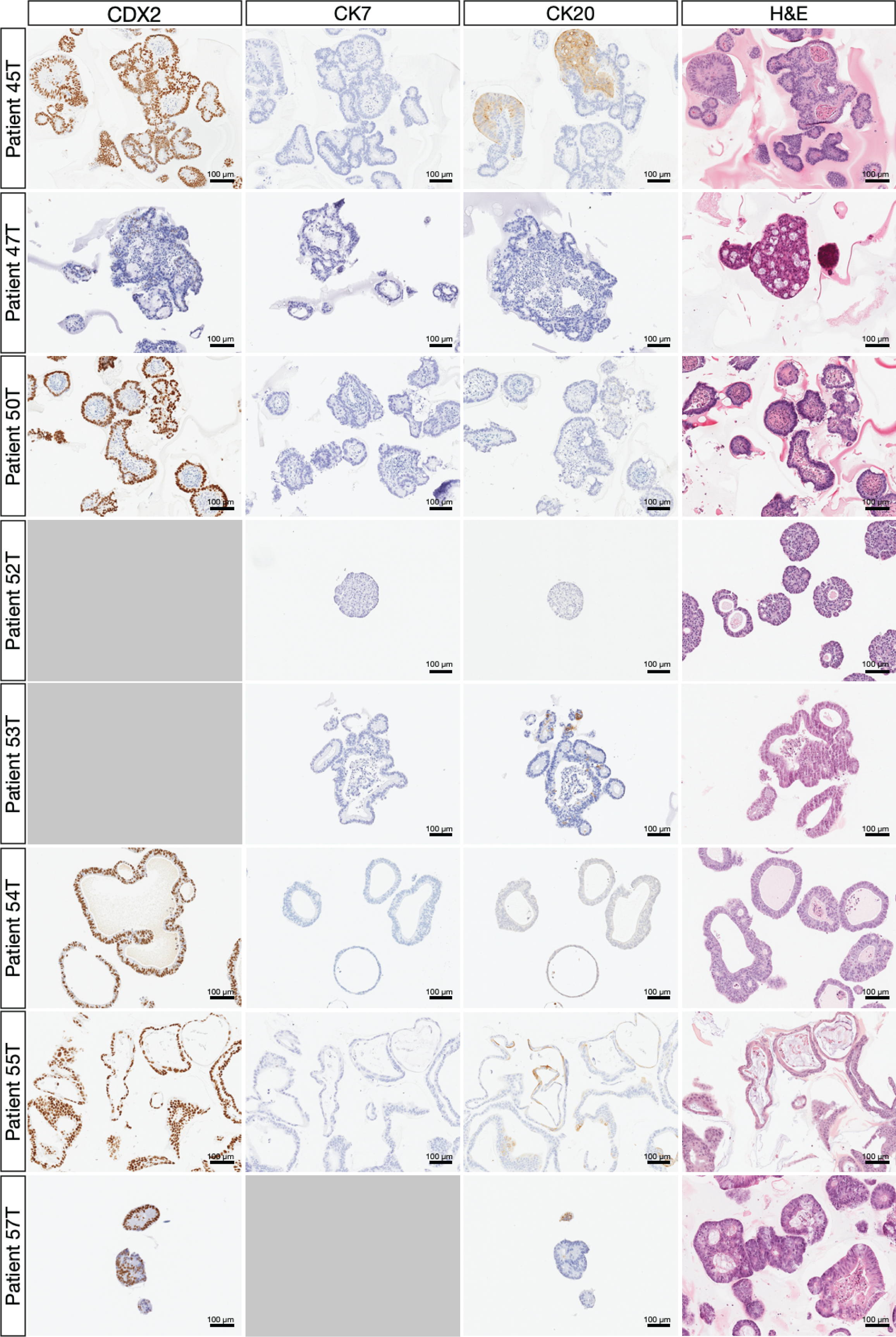

Supplement: Supplementary file 1 — Figure S1. Patient‐derived colorectal cancer organoids stained with markers used in the differential diagnosis of colorectal cancer, CDX2 (left), CK7 (second from left) and CK20 (second from right) as well as H&E staining (right). Scale bar, 100 μm. [file JGH-37-898-s002.zip › JGH_15818_Supplementary_Figure_1_Page2.png]

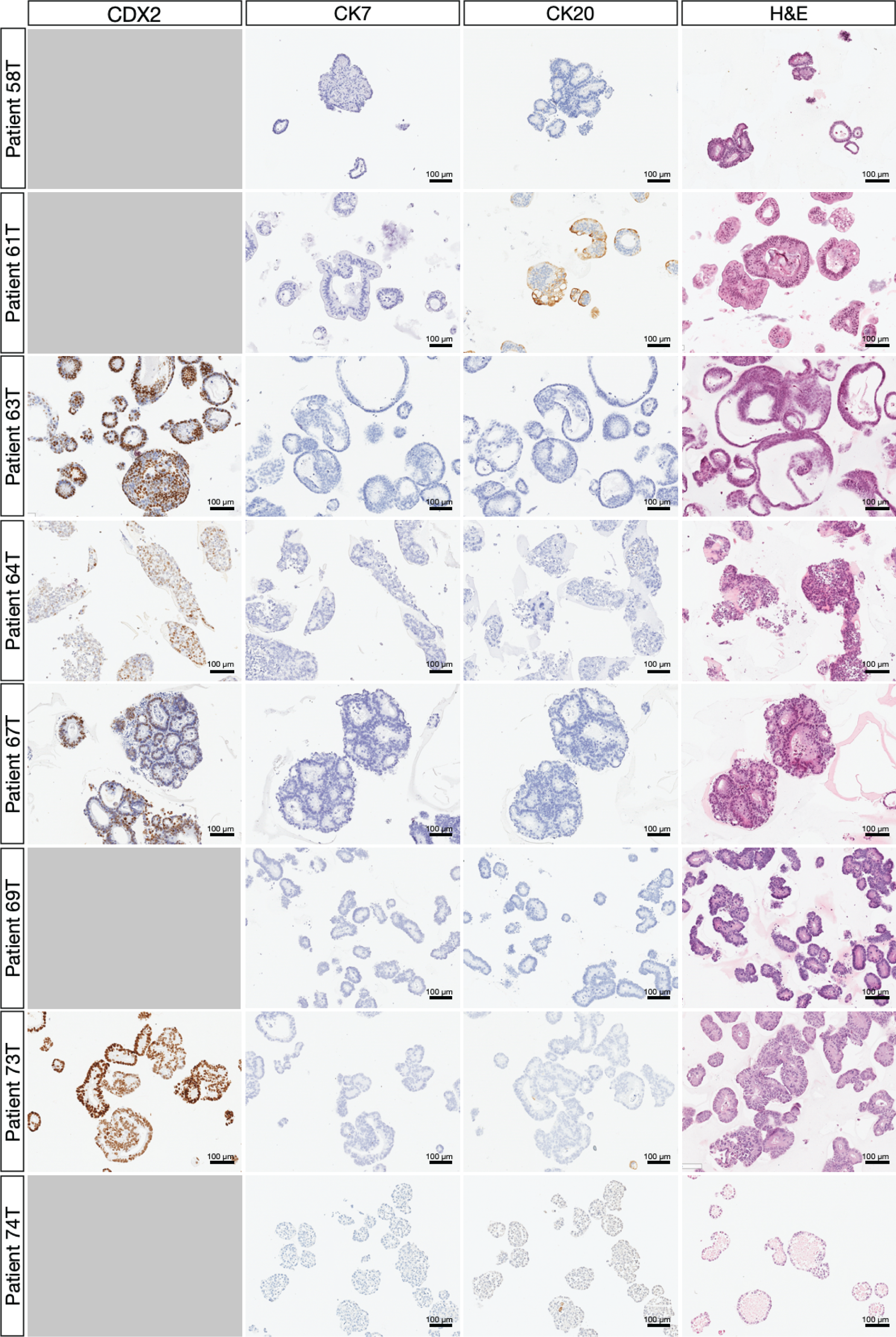

Supplement: Supplementary file 1 — Figure S1. Patient‐derived colorectal cancer organoids stained with markers used in the differential diagnosis of colorectal cancer, CDX2 (left), CK7 (second from left) and CK20 (second from right) as well as H&E staining (right). Scale bar, 100 μm. [file JGH-37-898-s002.zip › JGH_15818_Supplementary_Figure_1_Page3.png]

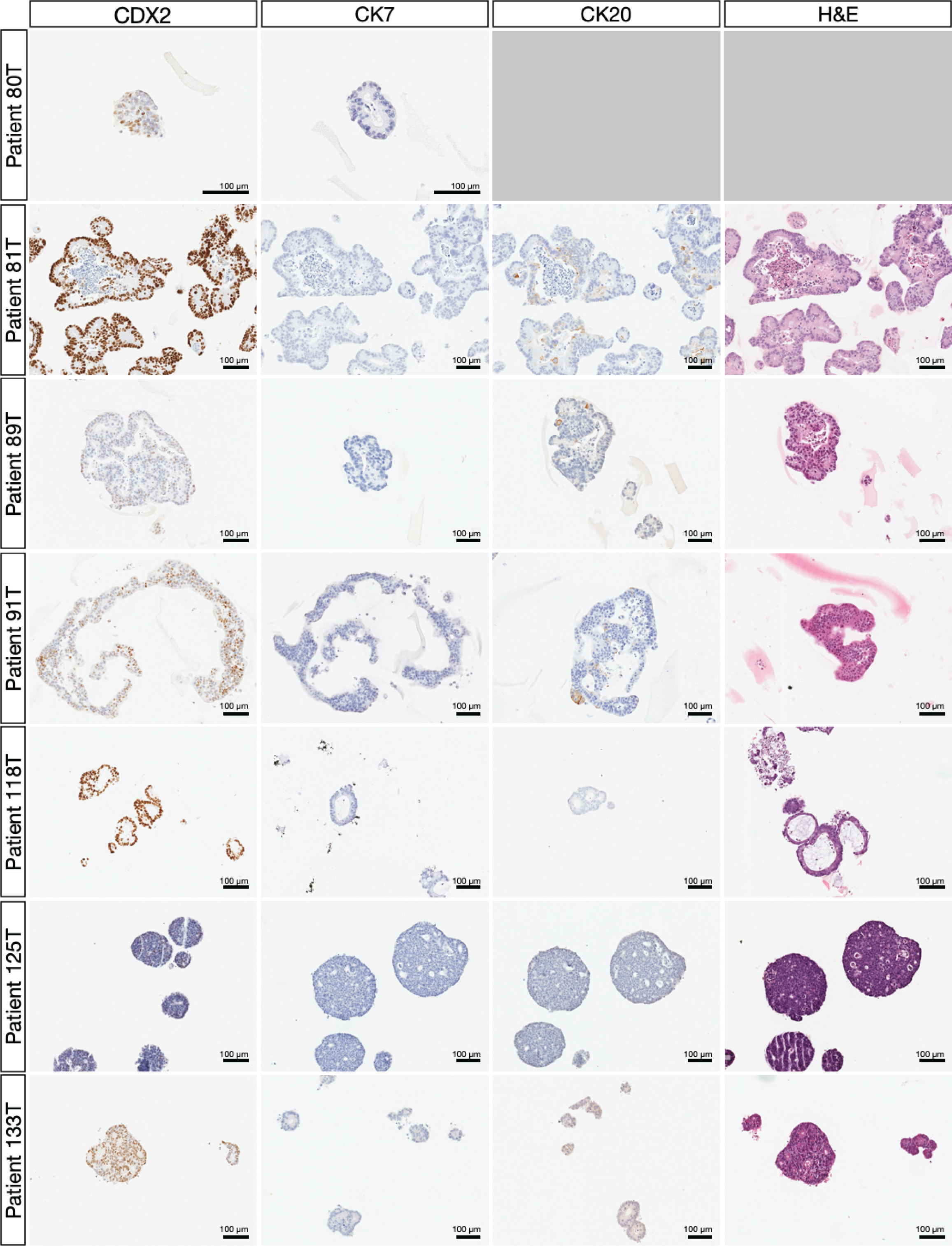

Supplement: Supplementary file 1 — Figure S1. Patient‐derived colorectal cancer organoids stained with markers used in the differential diagnosis of colorectal cancer, CDX2 (left), CK7 (second from left) and CK20 (second from right) as well as H&E staining (right). Scale bar, 100 μm. [file JGH-37-898-s002.zip › JGH_15818_Supplementary_Figure_1_Page4.png]

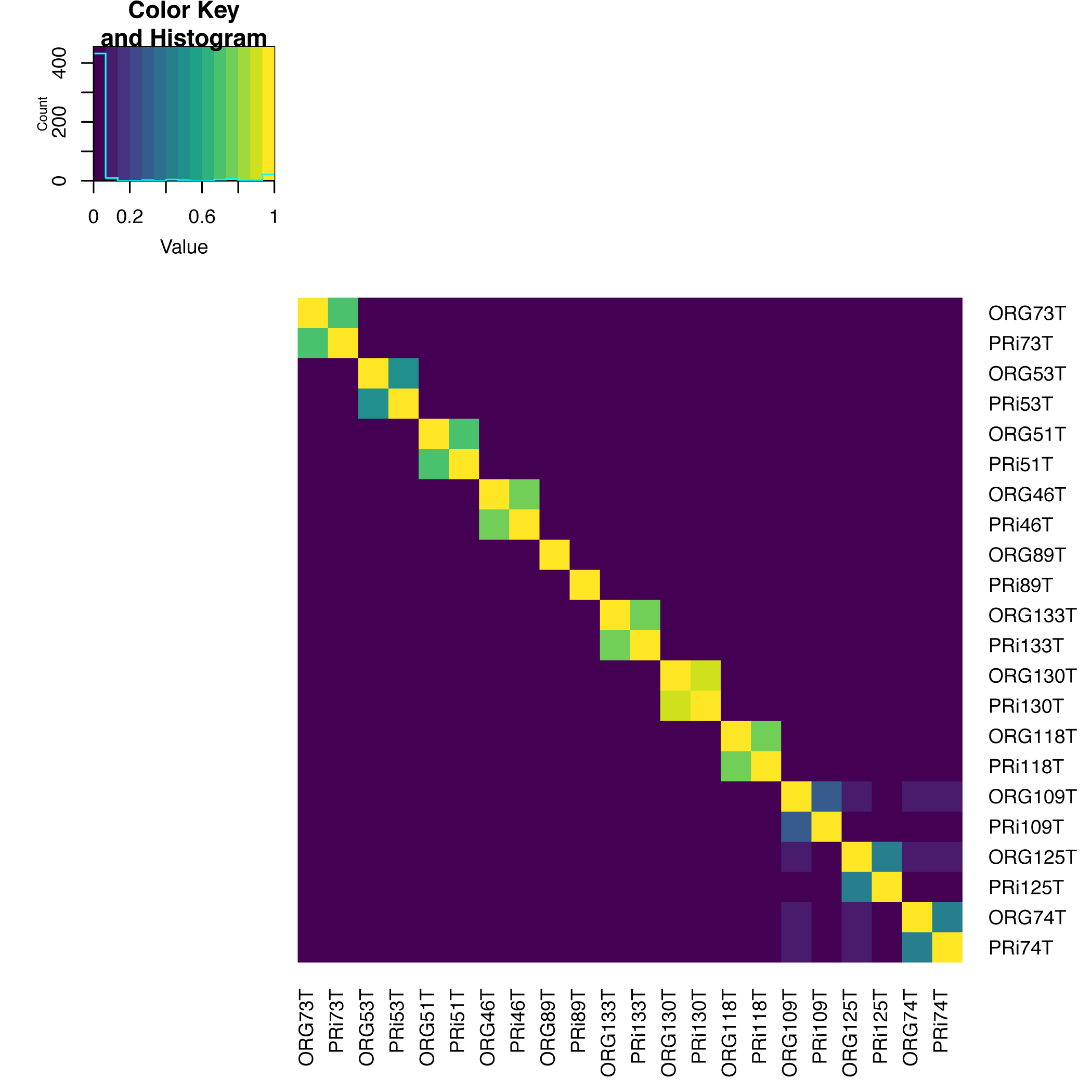

Supplement: Supplementary file 2 — Figure S2. Heat map of sample‐to‐sample distances comparing high‐confidence somatic variant calls for all PDCOs and primary tumor tissues sequenced in this study. The Jaccard distance between each pair of samples was calculated to verify that organoids had more somatic variants in common with their parental tumors than other organoid or tumor samples. [file JGH-37-898-s003.png]

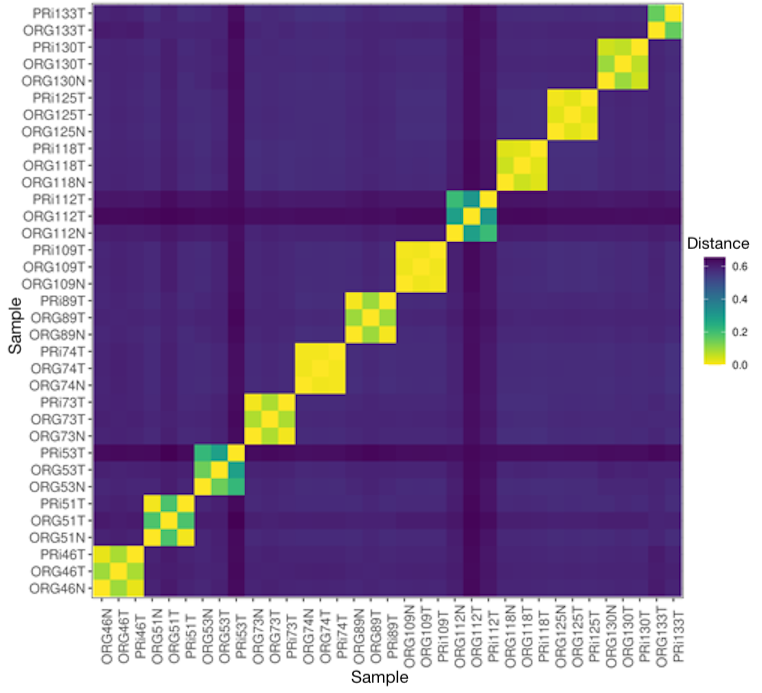

Supplement: Supplementary file 3 — Figure S3. Heat map of sample‐to‐sample distances comparing high‐confidence homozygous variant calls for all PDCOs, primary tumor tissue and normal tissues/organoids sequenced in this study. The Jaccard distance between each pair of samples was calculated to verify that samples from the same patient had more variants in common between them than in unrelated pairs of samples. [file JGH-37-898-s001.png]
