# Supplementary material for: Modeling colorectal cancer: A bio‐resource of 50 patient‐derived organoid lines
Source: J Gastroenterol Hepatol. 2022 Mar 10;37(5):898–907. doi: 10.1111/jgh.15818 (PMC10138743; doi:10.1111/jgh.15818)
Supplement: Supplementary file 4 — Table S1. Patient clinical characteristics for the patient‐derived cancer organoids. [file JGH-37-898-s004.docx]

| **Supplementary Table 1. Patient clinical characteristics for the patient-derived cancer organoids** | | | | | | | | | | | | | |
| --- | --- | --- | --- | --- | --- | --- | --- | --- | --- | --- | --- | --- | --- |
| **PDCO number** | **Primary tumor site** | **Age** | **Gender** | **Tumor type** | **Tumor grade** | **MMR status** | **T** | **N** | **M** | **Overall stage** | **Matched normal org (Y)** | **Matched met org (Y(n))** | **Site of metastasis** |
| **ORG1T** | sigmoid colon | 74 | M | adeno | moderate | normal | 3 | 0 | 0 | 2 |  |  |  |
| **ORG4T** | hepatic flexure | 91 | M | adeno | moderate | normal | 3 | 2 | 0 | 3 |  |  |  |
| **ORG7T** | ascending colon | 83 | M | adeno | well | normal | 3 | 2 | 0 | 3 |  |  |  |
| **ORG9T** | ascending colon | 72 | F | adeno | poor | dMMR MLH1/PMS2 absent | 3 | 0 | 0 | 2 |  |  |  |
| **ORG14T** | ascending colon | 82 | F | adeno mucinous | moderate | normal | 3 | 1 | 0 | 3 |  |  |  |
| **ORG18T** | ascending colon | 64 | M | adeno | moderate | normal | 2 | 1 | 0 | 3 |  |  |  |
| **ORG30T** | ascending colon | 86 | F | adeno mucinous | moderate | normal | 3 | 0 | 0 | 2 | Y |  |  |
| **ORG31T** | hepatic flexure | 55 | F | adeno | moderate | normal | 2 | 0 | 0 | 1 |  |  |  |
| **ORG36T** | rectosigmoid | 56 | F | adeno | poor | normal | 4 | 2 | 1 | 4 | Y |  |  |
| **ORG38T** | sigmoid colon | 84 | F | adeno | moderate | normal | 3 | 1 | 0 | 3 |  |  |  |
| **ORG45T** | ascending colon | 75 | M | adeno mucinous | moderate | normal | 3 | 0 | 0 | 2 |  |  |  |
| **ORG46T** | hepatic flexure | 74 | F | adeno | moderate | normal | 3 | 1 | 0 | 3 | Y |  |  |
| **ORG47T** | transverse colon | 79 | M | adeno | poor | dMMR MLH1/PMS2 absent | 3 | 0 | 0 | 2 |  |  |  |
| **ORG49T** | descending colon | 64 | F | adeno | moderate | normal | 3 | 1 | 0 | 3 |  |  |  |
| **ORG50T** | rectum upper third (> 12cm) | 70 | M | adeno | moderate | normal | 3 | 0 | 0 | 2 |  |  |  |
| **ORG51T** | rectum upper third (> 12cm) | 60 | M | adeno | moderate | normal | 3 | 1 | 0 | 3 |  |  |  |
| **ORG52T** | caecum | 72 | M | adeno | poor | dMMR MLH1/PMS2 absent | 3 | 0 | 0 | 2 |  |  |  |
| **ORG53T** | ascending colon | 81 | M | adeno | poor | normal | 3 | 1 | 0 | 3 |  |  |  |
| **ORG54T** | caecum | 66 | F | adeno | poor | normal | 3 | 2 | 0 | 3 |  |  |  |
| **ORG55T** | rectum upper third (> 12cm) | 70 | M | adeno | well | normal | 3 | 0 | 0 | 2 |  |  |  |
| **ORG57T** | sigmoid colon | 71 | M | adeno | moderate | normal | 2 | 0 | 0 | 1 |  |  |  |
| **ORG58T** | sigmoid colon | 75 | M | adeno | poor | normal | 2 | 0 | 0 | 1 |  |  |  |
| **ORG60T** | rectum lower third (< 8cm) | 78 | F | adeno | moderate | normal | 2 | 0 | 0 | 1 |  |  |  |
| **ORG61T** | transverse colon | 87 | F | adeno | moderate | normal | 3 | 0 | 0 | 2 |  |  |  |
| **ORG63T** | ascending colon | 79 | F | adeno | moderate | normal | 2 | 0 | 0 | 1 |  |  |  |
| **ORG64T** | caecum | 41 | F | adeno | poor | normal | 3 | 1 | 1 | 4 | Y | Y (3) | liver |
| **ORG67T** | rectum upper third (> 12cm) | 38 | M | adeno | moderate | normal | 3 | 2 | 1 | 4 | Y |  |  |
| **ORG69T** | ascending colon | 59 | F | adeno | moderate | dMMR MLH1/PMS2 absent | 3 | 0 | 0 | 2 | Y |  |  |
| **ORG73T** | rectum mid third (8-12cm) | 27 | M | adeno | poor | normal | 3 | 2 | 1 | 4 | Y | Y (1) | liver |
| **ORG74T** | descending colon | 66 | M | adeno | poor | dMMR MLH1/PMS2 absent | 4 | 2 | 1 | 4 | Y | Y (1) | peritoneum |
| **ORG76T** | rectum mid third (8-12cm) | 73 | M | adeno | moderate | normal | 2 | 0 | 0 | 1 | Y |  |  |
| **ORG77T** | rectum mid third (8-12cm) | 54 | M | adeno | well | normal | 1 | 0 | 0 | 1 |  |  |  |
| **ORG78T** | rectum upper third (> 12cm) | 55 | F | adeno | moderate | normal | 2 | 2 | 0 | 3 | Y |  |  |
| **ORG80T** | descending colon | 68 | M | adeno | moderate | normal | 2 | 0 | 0 | 1 | Y |  |  |
| **ORG81T** | rectosigmoid | 85 | F | adeno | moderate | normal | 2 | 0 | 0 | 1 |  |  |  |
| **ORG86T** | transverse colon | 80 | F | adeno | moderate | normal | 3 | 0 | 0 | 2 | Y |  |  |
| **ORG88T** | ascending colon | 84 | F | adeno mucinous | moderate | dMMR MLH1/PMS2/MSH6 absent | 3 | 0 | 0 | 2 | Y |  |  |
| **ORG89T** | rectosigmoid | 72 | M | adeno | moderate | normal | 3 | 2 | 1 | 4 | Y |  |  |
| **ORG91T** | transverse colon | 72 | F | adeno mucinous | moderate | normal | 3 | 2 | 1 | 4 | Y |  |  |
| **ORG92T** | caecum | 64 | F | adeno | poor | dMMR PMS2 absent | 3 | 1 | 0 | 3 | Y |  |  |
| **ORG95T** | hepatic flexure | 60 | F | adeno | moderate | normal | 3 | 1 | 0 | 3 | Y |  |  |
| **ORG104T** | caecum | 62 | M | adeno mucinous | well | normal | 3 | 1 | 0 | 3 | Y |  |  |
| **ORG109T** | transverse colon | 71 | F | adeno | poor | dMMR MLH1/PMS2 absent | 4 | 1 | 0 | 3 | Y |  |  |
| **ORG112T** | ascending colon | 53 | M | adeno | moderate | normal | 3 | 1 | 0 | 3 |  |  |  |
| **ORG115T** | rectosigmoid | 92 | M | adeno | moderate | normal | 4 | 0 | 0 | 2 | Y |  |  |
| **ORG117T** | rectosigmoid | 46 | F | adeno | moderate | normal | 3 | 0 | 0 | 2 | Y |  |  |
| **ORG118T** | caecum | 86 | M | adeno mucinous | moderate | normal | 4 | 1 | 1 | 4 | Y |  |  |
| **ORG125T** | ascending colon | 81 | F | adeno | poor | dMMR MLH1/PMS2 absent | 3 | 1 | 0 | 3 | Y |  |  |
| **ORG130T** | rectum upper third (> 12cm) | 88 | F | adeno | moderate | normal | 3 | 1 | 0 | 3 | Y |  |  |
| **ORG133T** | sigmoid colon | 82 | M | adeno | poor | normal | 3 | 2 | 0 | 3 | Y |  |  |
