# Supplementary material for: Modeling colorectal cancer: A bio‐resource of 50 patient‐derived organoid lines
Source: J Gastroenterol Hepatol. 2022 Mar 10;37(5):898–907. doi: 10.1111/jgh.15818 (PMC10138743; doi:10.1111/jgh.15818)
Supplement: Supplementary file 5 — Table S2. Somatic variants identified in patient‐derived cancer organoids. [file JGH-37-898-s005.docx]

| Supplementary Table 2. Somatic variants identified in patient-derived cancer organoids | | | | | | | | |
| --- | --- | --- | --- | --- | --- | --- | --- | --- |
| PDCO | **Chr** | **position** | **reference** | **variant** | **Gene** | **effect** | **Matched in PriT?** |  |
| ORG51T | 5 | 112173917 | C | T | APC | nonsense | Y |  |
|  | 17 | 7578535 | T | C | TP53 | nonsynonymous | Y |  |
| ORG125T | 5 | 112175614 | A | -3 | APC | nonsynonymous | Y |  |
|  | 7 | 140453136 | A | T | BRAF | nonsynonymous | Y |  |
|  | 17 | 7577094 | G | A | TP53 | nonsynonymous | Y |  |
|  | 17 | 7578457 | C | T | TP53 | nonsynonymous | Y |  |
| ORG130T | 5 | 112174112 | G | T | APC | nonsense | Y |  |
|  | 17 | 7578236 | A | G | TP53 | nonsynonymous | Y |  |
| ORG133T | 5 | 112174425 | A | -4 | APC | frameshift | Y |  |
|  | 5 | 112175575 | A | -1 | APC | frameshift | Y |  |
|  | 12 | 25398285 | C | A | KRAS | nonsynonymous | Y |  |
|  | 17 | 7577102 | C | -9 | TP53 | nonsynonymous | Y |  |
| ORG46T | 3 | 178916891 | G | A | PIK3CA | nonsynonymous | Y |  |
|  | 5 | 112162922 | C | A | APC | nonsynonymous | Y |  |
|  | 5 | 112175675 | C | A | APC | frameshift | Y |  |
|  | 7 | 140453136 | A | T | BRAF | nonsynonymous | Y |  |
|  | 17 | 7577141 | C | A | TP53 | nonsynonymous | Y |  |
| ORG53T | 3 | 178936092 | A | G | PIK3CA | nonsynonymous | N |  |
|  | 5 | 112128191 | C | T | APC | nonsense | Y |  |
|  | 17 | 7577538 | C | T | TP53 | nonsynonymous | Y |  |
| ORG109T | 5 | 112175925 | C | -1 | APC | frameshift | N |  |
|  | 7 | 140453136 | A | T | BRAF | nonsynonymous | Y |  |
|  | 17 | 7577121 | G | A | TP53 | nonsynonymous | N |  |
|  | 17 | 7578440 | T | C | TP53 | nonsynonymous | Y |  |
| ORG112T | 5 | 112154972 | G | A | APC | nonsynonymous | Y |  |
|  | 17 | 7577506 | C | A | TP53 | nonsynonymous | Y |  |
| ORG73T | 3 | 178927980 | T | C | PIK3CA | nonsynonymous | Y |  |
|  | 5 | 112173830 | G | +A | APC | frameshift | Y |  |
|  | 5 | 112175523 | G | -1 | APC | frameshift | Y |  |
|  | 12 | 25398285 | C | A | KRAS | nonsynonymous | Y |  |
|  | 17 | 7579358 | C | A | TP53 | nonsynonymous | Y |  |
| ORG74T | 3 | 178936091 | G | A | PIK3CA | nonsynonymous | Y |  |
|  | 7 | 140453136 | A | T | BRAF | nonsynonymous | Y |  |
| ORG89T | 5 | 112175490 | C | T | APC | nonsynonymous | N |  |
|  | 17 | 7577548 | C | T | TP53 | nonsynonymous | N |  |
| ORG118T | 5 | 112154967 | T | +A | APC | frameshift | Y |  |
|  | 5 | 112175639 | C | T | APC | nonsense | Y |  |
|  | 7 | 140453136 | A | T | BRAF | nonsynonymous | Y |  |

PriT = patient matched primary tumor tissue
